# Fabrication of interconnected porous Ag substituted octacalcium phosphate blocks based on a dissolution-precipitation reaction

**DOI:** 10.1007/s10856-022-06672-5

**Published:** 2022-05-31

**Authors:** Yuki Sugiura, Masanori Horie

**Affiliations:** Health and Medical Research Institute, National Institute of Advanced Industrial Science and Technology (AIST), 2217-14 Hayashi-cho, Takamatsu, Kagawa, 761-0395 Japan

## Abstract

Here, we introduce Ag substituted octacalcium phosphate (OCP-Ag) blocks with interconnected porous structure and sufficient mechanical strength as bone substitute (i.e., foam). We employed a two-step process for fabrication, which includes a setting reaction for acidic calcium phosphate granules using an acidic phosphate solution and a phase conversion process via dissolution-precipitation method in cocktail ((NH_4_)_2_HPO_4_-NH_4_NO_3_-NaNO_3_-AgNO_3_) solutions. The Ag contents in the fabricated OCP-Ag foams were 0.08–0.15 at%, which were sufficient in exhibiting contact antibacterial ability. The mechanical strength and porosity of the OCP-Ag foams were about 0.5 MPa and 70%, respectively. These values were sufficient for the application of the OCP-Ag foams as bone substitute.

**Figure:**
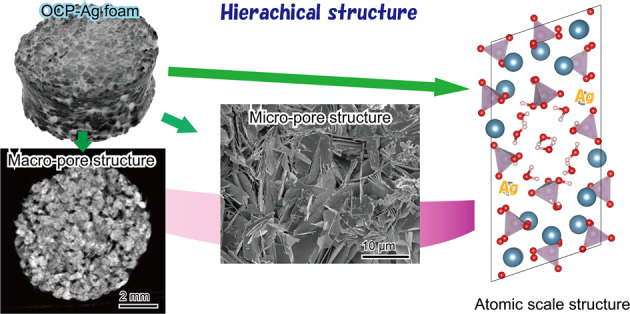
Graphical abstract

Graphical abstract

## Introduction

Calcium phosphates blocks that exhibit osteo-conductivity are commonly used biomaterials for bone defect reconstruction in orthopedic and oral surgery. The attachment of an interconnected porous structure (i.e., foam) to a scaffold has been an effective way to accelerate the replacement of the scaffold to bone, because the attachment occurs only on the surface of the scaffold [1–3]. Foams have been known to enhance tissue response because their interconnected pores allow cells and tissues to penetrate the interior of the scaffold; thereby, facilitating the supply of vital nutrients by vascular ingrowth [1, 4–6].

It is important to regulate infection during bone defect regeneration [7–9]. Once an infection occurs, it often leads to serious clinical cases. An infection in bone substitutes for bone defect reconstruction has been a serious risk factor because the substitutes are implanted inside a periosteum, which is a barrier against bone infection [10]. Meanwhile, in cases of open fracture, control of infection is still an important clinical problem.

We have been working on octacalcium phosphate [OCP: (Ca_8_(PO_4_)_4_(HPO_4_)_2_·5H_2_O)] based materials for applications in bone substitution materials and attachment of antibacterial ability. OCP is a main component of immature bone and exhibits excellent biocompatibility [1, 11–17]. Based on our evaluation, taking advantage of the low symmetrical crystal structure (*P*-1) in OCP enables the doping of various elements and molecules into OCP crystal structures [18–22]. Recently, we doped silver (Ag) into an OCP crystal structure as replacing Ca in the conjugated site of *P*5 PO_4_ (OCP-Ag) powders and blocks [23, 24]. The OCP-Ag exhibited both excellent contact antibacterial ability and low cytotoxicity for osteoblast.

The application of OCP-Ag in bone substitutes is a valuable way to attach interconnected porous structures that allow tissue penetration. In this study, we investigated OCP-Ag blocks, which have stimulatory interconnected porous structure, appropriate size, and mechanical strength for applications in bone substitutes.

## Experimental methods

### Fabrication of precursor acidic calcium phosphate ceramic foams

All reagents were purchased from Fujifilm Wako Pure Chem Inc. (Osaka, Japan), except for β-tricalcium phosphate [β-TCP: (Ca_3_(PO_4_)_2_)], which was purchased from Taihei Chemical Industrial Inc. (Osaka, Japan).

The details of precursor ceramic foams were described in our previous study [25]. Briefly, acidic calcium phosphate granules were fabricated as analogs of the dental brushite cement setting reaction. Monocalcium dihydrogen phosphate monohydrate [MCPM: Ca(H_2_PO_4_)_2_·H_2_O] and β-TCP were mixed at a molar ratio of 1:1 using an agate mortar and pestle. 1 g of the mixture was transferred into a rotary pan-type granulator PZ-01R from As One Co. (prefecture, Japan) with stirring at 40 rpm. 1.0 mL of distilled water was then added as a spray to obtain granules of acidic calcium phosphate. The resulting mixture was stirred continuously for 10 min and then placed in a dry oven at 40 °C overnight. The dried mixture was separated using an automatic sieve into five granule sizes of <100, 100–250, 250–500, 500–1000, and 1000–2000 μm. All separated sizes (except <100 μm sizes) were used for further treatment.

An acidic phosphate solution for the setting reaction was prepared from 1 mol/L phosphoric acid (H_3_PO_4_), which was saturated with MCPM. This solution was diluted by distilled water for the setting reaction. The sieved granules were placed into silicon rubber molds with dimension of *φ*6 × 3 mm. Then, 50 μL of a diluted acidic phosphate solution was dropped onto the sieved granules, which were immediately packed for the setting reaction. We employed 0.4, 0.5, 0.7, and 0.7 mol/L H_3_PO_4_ (saturated with MCPM) to the 100–250, 250–500, 500–1000, and 1000–2000 μm granules, respectively for the setting reactions. After a few minutes, the granules were dried in an oven at 60 °C for several hours. The set granules were stored at 40 °C for further reactions.

### Phase conversion process of an acidic calcium phosphate foam in Ag containing solutions

We first prepared mother solutions of 5 mol/L NaNO_3_, 200 mmol/L AgNO_3_, and 2 mol/L (NH_4_)_2_HPO_4_-NH_4_NO_3_. The pH of the 2 mol/L (NH_4_)_2_HPO_4_-NH_4_NO_3_ solution was adjusted to 9.0 by addition of 25% ammonia in water. Appropriate volumes of mother solutions were mixed to prepare a final reacting solution that contained 1.0 mol/L-NaNO_3_, 20 mmol/L AgNO_3_, and 1 mol/L (NH_4_)_2_HPO_4_-NH_4_NO_3_. The initial pH of the final solution was about 8.5.

Ten pieces of acidic calcium phosphate foams were immersed into a reaction solution and tightly sealed by Teflon^®^ tapes from company (prefecture country). The resulting sample was stored at 70 °C for 2 days. Then, the treated acidic calcium phosphate foams were washed by distilled water several times and dried overnight at 40 °C.

### Characterization

The sample morphology was studied by field emission scanning electron microscopy using a JSM-6700F from JEOL Co. (prefecture, Japan) with an accelerating voltage of 3 kV after coating a sample with osmium. The compositions of the samples were determined by X-ray diffraction (XRD) using a MiniFlex600 from Rigaku Co. (prefecture, Japan) at 40 kV and 15 mA. A sample was processed through crushing using an agate mortar and pestles. The diffraction angle was continuously scanned in a 2° range of 3°–70°.

The Ca^2+^, P (as PO_4_^3−^), and Ag+ ion concentrations in the samples were determined by inductively coupled plasma atomic emission spectroscopy (ICP-AES) using a 5110VDV from Agilent Technology Co. (prefecture, Japan). The samples were dissolved in 2% HNO_3_ prior to ICP-AES.

The mechanical strengths of the samples were evaluated in terms of DTS. After measuring the diameters and heights using a micrometer MDC-25MU from Mitutoyo Co. (prefecture, Japan), the samples were crushed using a universal testing machine AGS-J from Shimadzu Co. (prefecture, Japan) at a constant crosshead speed of 1 mm/min. The average values and standard deviations of the DTS (*σ*) were calculated from the results of the breaking strength (*P*) of five specimens (*n* = 5) by Eq. (1).

1 $$\sigma = 2P/\pi dl$$

where, *d* and *l* are the diameter of the specimen and length, respectively.

The macroporosity (*P*_m_) of the samples was calculated using the bulk density method (Eq. (2)).

2 $$P_m = \left( {2.67 \times 10^3 - w/\pi dl} \right) \times 100$$

Here, *w* and 2.67 × 10^3 ^kg/m^3^ are the weight of a sample and density of solid OCP, respectively. The average values and standard deviations of the porosity were calculated using the results from five samples (*n* = 5). Analysis of the internal morphology was performed using a quantitative three-dimensional evaluation program, which is included in the microcomputed tomography system (microCT) Skyscan 1075 KHS from Kontich (state, Belgium) with a source voltage (69 kV), source current (149 μA), and Al filter (0.5 mm). The voxel resolution was 9 μm.

## Result and discussions

**Fig. 1:**
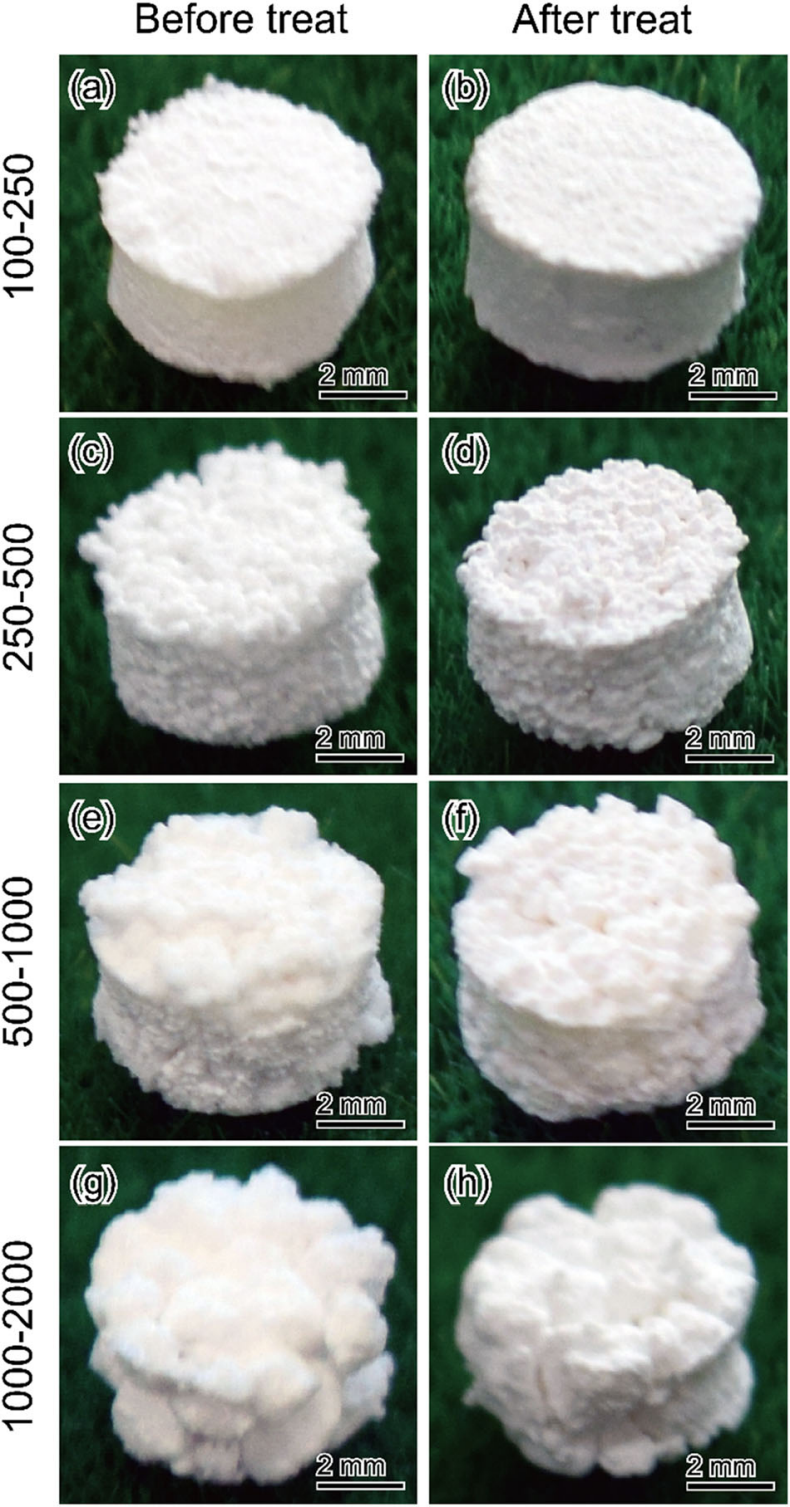
Representative photographs of acidic calcium phosphate foams before (**a**–**d**) and after (**e**–**h**) immersion

Representative typical photographs of the acidic calcium phosphate foam before and after immersion are shown in Fig. 1. The entire structure of all the foam samples were maintained after immersion. The color of the samples was unchanged throughout the immersion process.

**Fig. 2:**
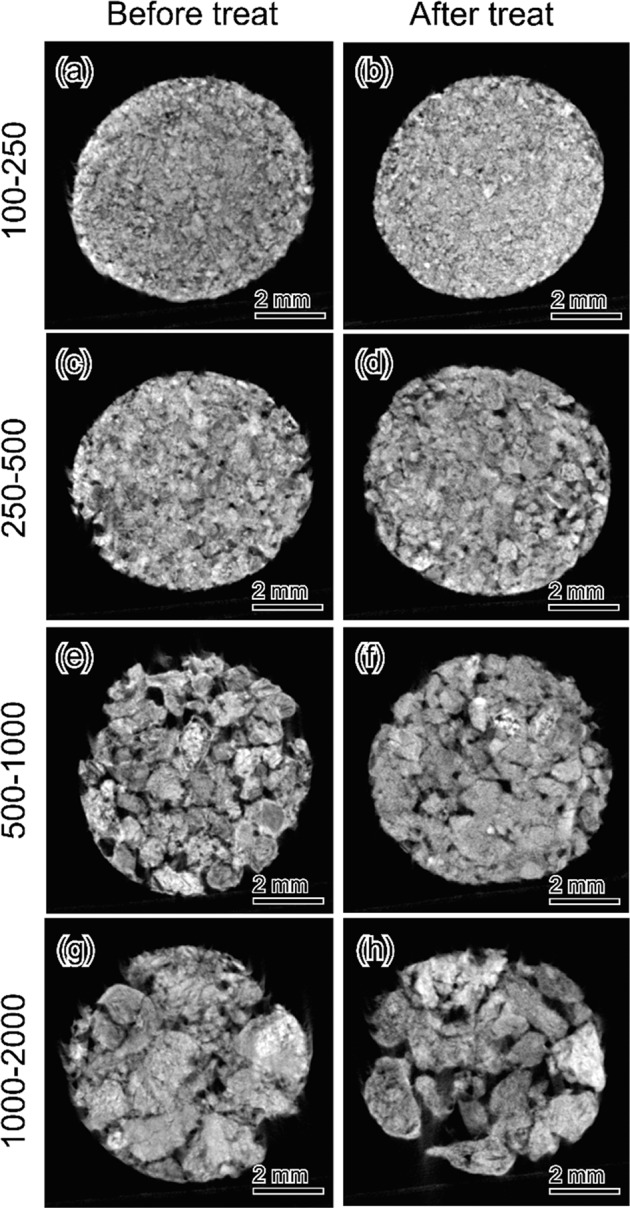
Representative cross-sectional microCT images of acidic calcium phosphate foams before (**a**–**d**) and after (**e**–**h**) immersion

Representative microCT images of the acidic calcium phosphate foam before and after immersion are shown in Fig. 2. The inner and entire structures of the samples were maintained. Therefore, the interconnectivity formed by the granules setting were maintained throughout the immersion processes.

**Fig. 3:**
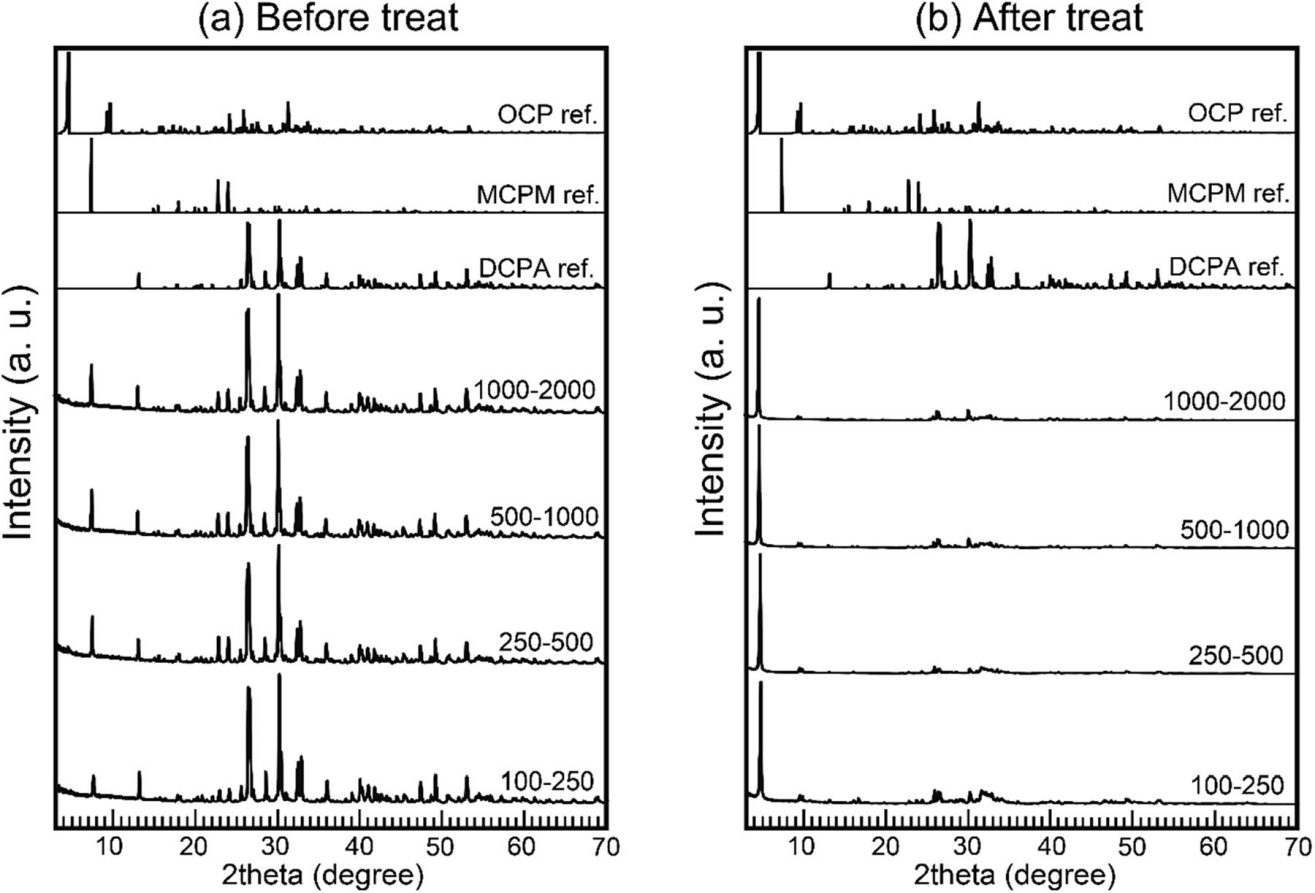
XRD patterns of acidic calcium phosphate before (**a**) and after **b** immersion. DCPA, MCPM, and OCP reference patterns were included to facilitate comparisons

The shapes of the samples were maintained throughout the immersion process using the Ag containing solution. The phases of the samples were studied to show the feasibility of the immersion process. The XRD patterns of the foams before and after immersion are shown in Fig. 3. Before immersion, the acidic calcium phosphate foams consisted mainly of calcium hydrogen phosphate anhydrate [DCPA: CaHPO_4_] and MCPM. After immersion, the treated foams became monophasic OCP.

**Fig. 4:**
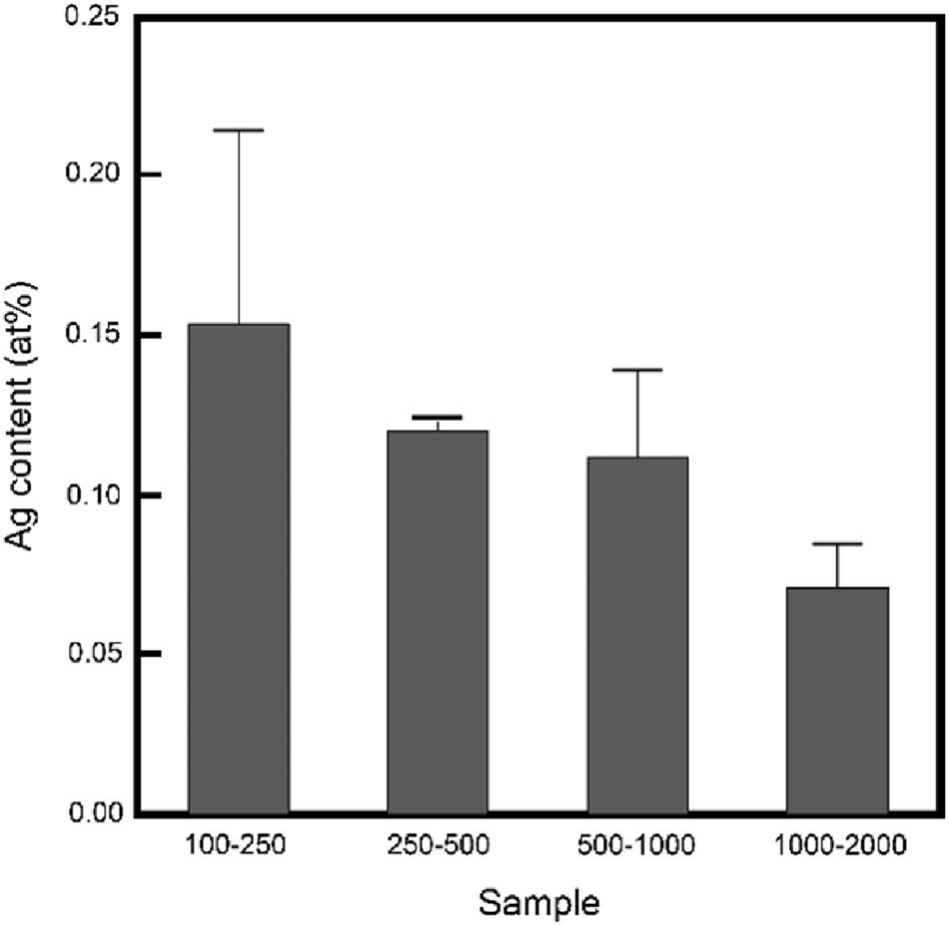
Ag contents in OCP-Ag foams as a function of initial granule sizes

The Ag contents in the acidic calcium phosphate foams after immersion are summarized in Fig. 4. The samples contained 0.08–0.15 at% Ag. The Ag content decreased with the increase in the granule sizes. Note, the Ag contents in the treated samples were sufficient for exhibiting contact antibacterial ability (>0.05 at%) in OCP [24]. Based on the XRD and elemental analysis results obtained for the treated samples, the acidic calcium phosphate foam after immersion were called as OCP-Ag foam.

**Fig. 5:**
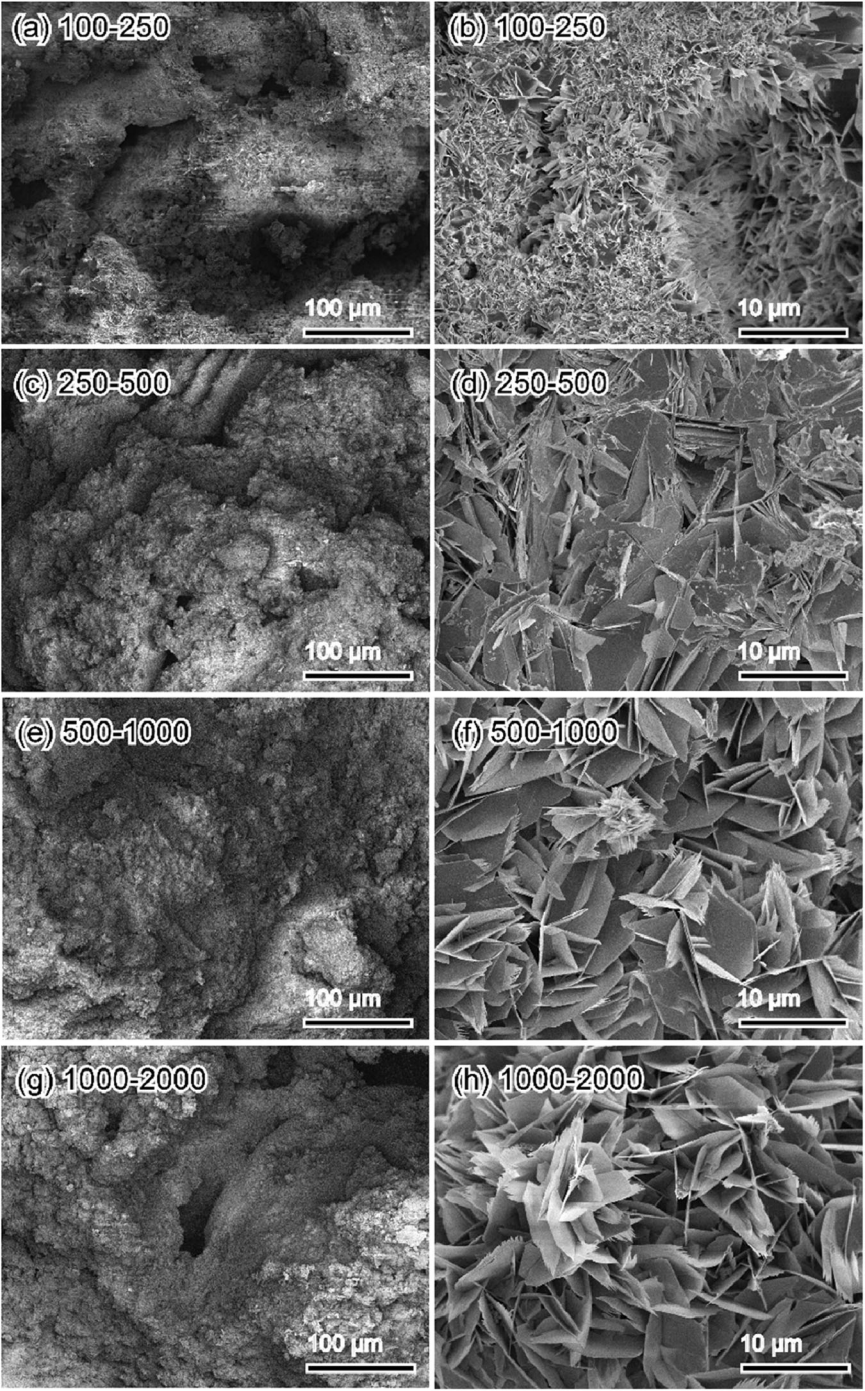
Representative SEM micrographs of OCP-Ag foams with sizes of (**a**, **b**) 100–250, **c**, **d** 250–500, **e**, **f** 500–1000, and (**g**, **h**) 1000–2000 μm

**Fig. 6:**
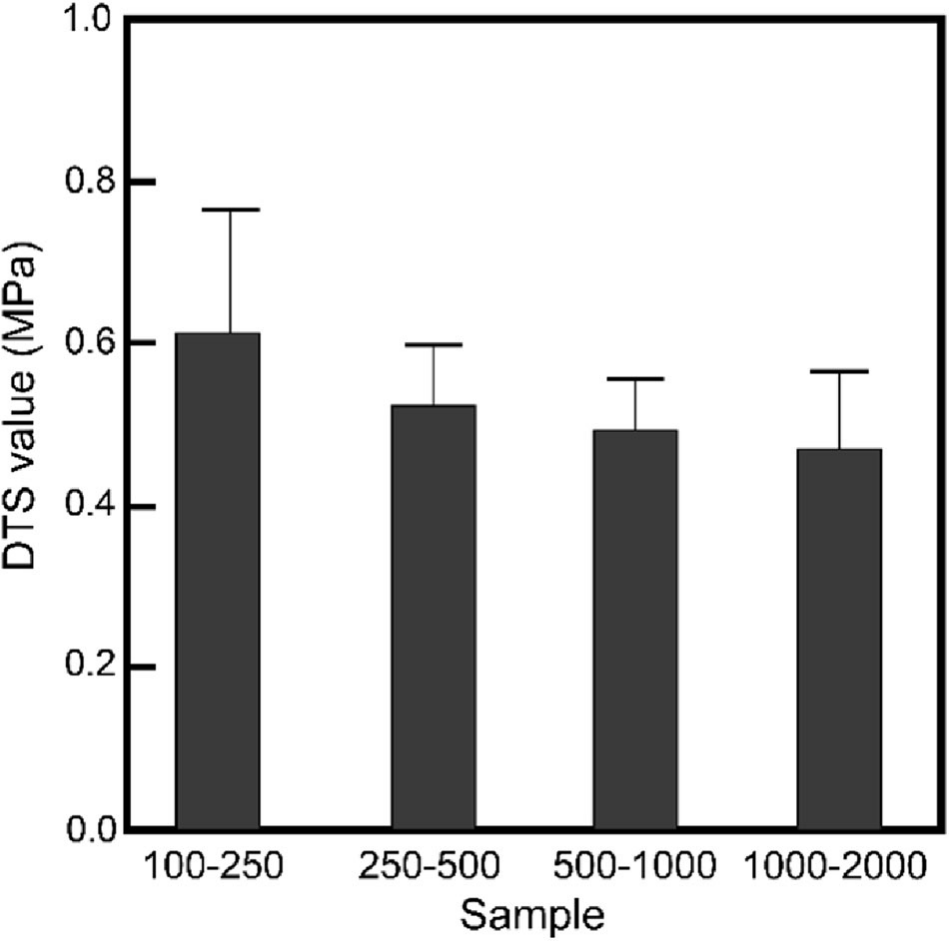
DTS values obtained for the OCP-Ag foams as a function of initial granule sizes

**Fig. 7:**
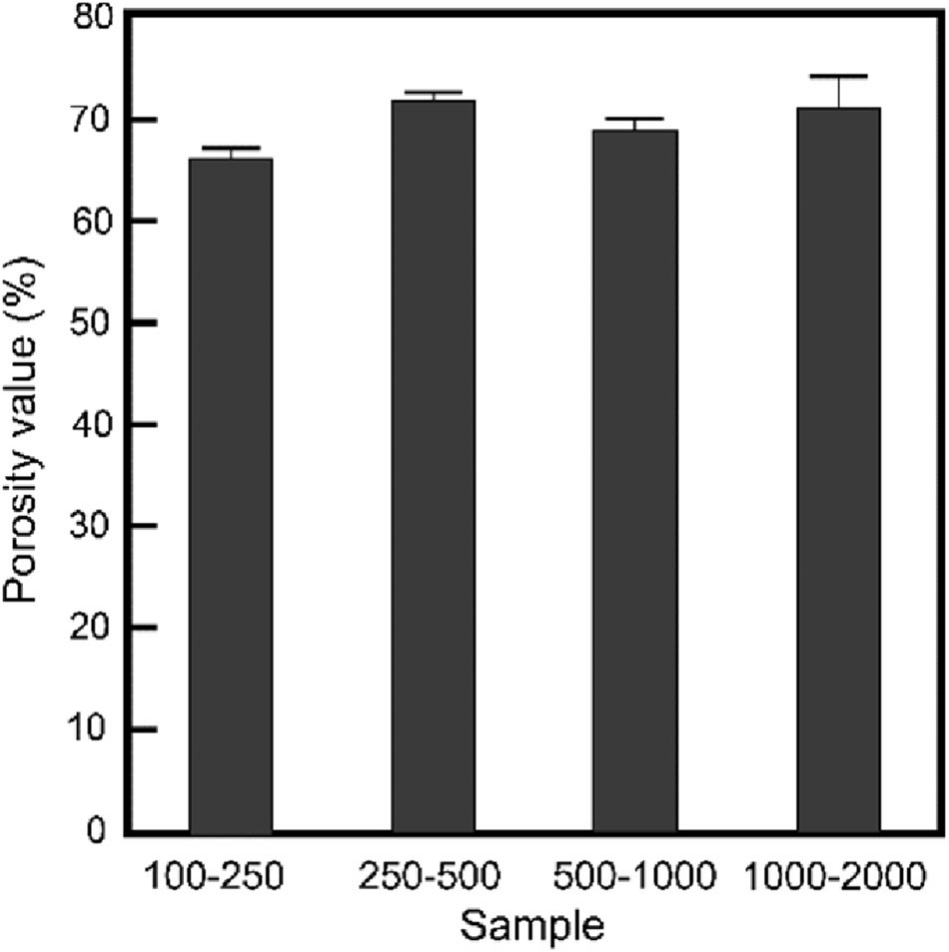
Porosity values obtained for the OCP-Ag foams as a function of initial granule sizes

The fine structure of the OCP-Ag foam is shown in Fig. 5. The entire structures inside observed by SEM and microCT were consistent. In high magnification images, the surface of each granule was covered closely by plate-like crystals with sizes of around 10 μm. The sizes of plate-like crystals increased with the increase in the size of the granules. The mechanical strength and porosity of OCP-Ag, Na foams were essential factors for applying them as bone substitute. Figures 6 and 7 showed the mechanical strength and porosities of OCP-Ag, Na foams, respectively. Although slightly decrease of DTS values was observed as increase of granule sizes, the DTS values of all samples were above 0.5 MPa that were enough values for implantation. The porosities of all samples were above 60% that were enough values for interconnectivity. Note, the porosity of samples obtained by mass-volume methods seemed to be much higher than the observed results by SEM. Therefore, we considered that many microscopic pores also formed in the fabricated OCP foams, especially, each settle granules.

The results suggested that the OCP-Ag foams were fabricated clearly from acidic calcium phosphate foams in an Ag containing solution via dissolution-precipitation reactions. Here, we employed an ionic insertion method, which was established by our previous study, for Ag substitution into OCP foams [26]. For the ionic insertion method, the dispersed cations in the reacting solution and weak basicity of the phosphate solutions were key for optimal substitution. The Ag^+^ was precipitated likely as Ag_3_PO_4_ in a weak basic phosphate solution. Co-existing NH_4_^+^ (or NH_3_) enabled the dispersion of Ag^+^ ions in weak basic phosphate solutions as [Ag(NH_3_)_2_]^+^ form. Furthermore, Na^+^ ion as OCP inducer co-existed in the reaction solutions [27, 28]. Thus, OCP foams with substituted Ag were fabricated successfully. Although the relationship between Na^+^ and Ag^+^ in OCP substitution was competition, Ag^+^ could be substituted above the threshold values [29].

The obtain results clearly indicated that OCP-Ag foams could be fabricated from acidic calcium phosphate foam through Ag containing solution via dissolution-precipitation reactions. In this study, for Ag substitution into OCP foams, we employed ionic insertion method which established our previous studies [26]. For ionic insertion method, how dispersed purpose cations in reacting solution, weak basic phosphate solutions, was the key for optimal substitution. Then, in the case of Ag, Ag^+^ was likely to precipitate as Ag_3_PO_4_ in weak basic phosphate solutions. Co-existing NH_4_^+^ (or NH_3_) in solutions enabled to disperse Ag^+^ ion in weak basic phosphate solutions as [Ag(NH_3_)_2_]^+^ form. Furthermore, Na^+^ ion, as OCP inducer, was also co-existing in reacting solutions [27, 28]. Thus, OCP foams could be fabricated and could be substituted Ag into OCP consisted of OCP foam. Although the relationships of Na^+^ and Ag^+^ for OCP substitution was competition, Ag could be substituted above threshold values [29].

The pore sizes of the foams were dominated by tissue penetration [30, 31]. The advantages of the proposed method for controlling pore sizes was the simplicity in changing the granules sizes in the setting reactions. A suitable precursor foam was fabricated successfully through one step (i.e., acidic calcium phosphate solution treatment). We note that the mechanical strength of the prepared OCP-Ag foams was significantly lower than the OCP foam without Ag. We considered that the size of the plate-like crystals and their interlocking processes on the surfaces of granules dominated the mechanical strength of the prepared materials.

## Conclusion

OCP-Ag foams with sufficient Ag contents (>0.05 at%) for exhibiting contact antibacterial ability and mechanical strength (>0.5 MPa in DTS) were fabricated successfully from settled acidic calcium phosphate granules, which were produced via a dissolution-precipitation reaction in weak basic phosphate solution with Ag ions. The change in the granule sizes allowed the pore size control of the OCP-Ag foam.
